# The Importance of the Taller-than-Wide Feature and the Dimensions of Focal Thyroid Lesions in Assessing the Risk of Malignancy

**DOI:** 10.3390/diagnostics16142193

**Published:** 2026-07-14

**Authors:** Marta Zuzanna Ciechomska, Dorota Szydlarska, Andrzej Śliwczyński

**Affiliations:** 1 Department of Family Medicine, National Medical Institute of the Ministry of the Interior and Administration, 02-507 Warsaw, Poland; dorota.szydlarska@pimmswia.gov.pl; 2 Department of Family Medicine, Medical University of Warsaw, 02-091 Warsaw, Poland; 3 Faculty of Health Sciences, WSEI University, 00-824 Warsaw, Poland; andrzej.sliwczynski@pimmswia.gov.pl

**Keywords:** thyroid nodules, ultrasound, risk stratification, taller-than-wide shape, malignancy risk, fine-needle aspiration biopsy, EU-TIRADS

## Abstract

**Background/Objectives:** A taller-than-wide shape is considered a suspicious ultrasound feature in the assessment of thyroid nodules. Although previous studies indicate an association with malignancy, its predictive value may vary depending on the population, methodology and measurement criteria. This study aimed to evaluate the diagnostic significance of the TTW feature and selected nodule dimensions in predicting malignancy risk. **Methods:** A retrospective observational study was conducted including 367 patients with thyroid nodules referred for fine-needle aspiration biopsy. Nodule height, width and the height-to-width ratio were assessed and compared with cytological outcomes according to the Bethesda classification. Spearman rank correlation and logistic regression analyses were performed, and the predictive value of the TTW ratio was evaluated using receiver operating characteristic analysis. **Results:** A statistically significant but weak positive correlation was observed between Bethesda category and both nodule height and the product of height and width (*p* < 0.05; r < 0.3). No significant association was found between the TTW ratio and lesions requiring further clinical evaluation and potential surgical management (OR = 1.03; *p* = 0.931). The area under the ROC curve for the TTW ratio was 0.497, indicating limited discriminatory performance between benign and potentially malignant lesions. **Conclusions:** In this cohort, the TTW feature showed limited predictive value in assessing malignancy risk. It should not be used as a standalone criterion for biopsy or surgery, but interpreted as part of a comprehensive ultrasound-based risk assessment together with cytological findings. Further studies in larger populations and with standardized ultrasound measurement protocols are warranted.

## 1. Introduction

Thyroid nodules are solid, liquid, or mixed-type lesions; while some of them may be palpable on physical examination, many others remain clinically undetectable and are diagnosed by ultrasound (US). While the lesions are usually benign, international data indicate that malignancies account for 5–15% of cases [1,2]. Within the Polish population, thyroid nodules pose a significant problem in daily clinical practice as their prevalence can be as high as 50–60% [3]. Thyroid ultrasound is widely recognized as the primary imaging modality [4,5,6,7] providing information about the size, shape, location, and morphological structure of the thyroid gland, thus facilitating identification of focal lesions [8]. The advantages of ultrasound diagnostics include high sensitivity, non-invasiveness, reproducibility, and no requirement for special patient preparation. The American Thyroid Association reports that nodular lesions are found on ultrasound in 19–67% of subjects, with thyroid carcinomas accounting for 5–15% of that group [9]. The first standardized criteria for ultrasound evaluation of thyroid nodules were introduced as part of the Thyroid Imaging Reporting and Data System (TI-RADS) [10,11]. Ultrasound features assessed in thyroid nodules include size, composition, echogenicity, shape, margins, calcifications, vascularity, and orientation, including the taller-than-wide (TTW) feature. These characteristics are used to guide further clinical management, including fine-needle aspiration biopsy (FNAB), surgical treatment, or active surveillance. In Poland, the risk of malignancy of focal thyroid lesions is stratified using the EU-TIRADS-PL system published in 2022 by the Polish Society of Endocrinology [12] and based on the principles of the European EU-TIRADS system [8]. Several suspicious ultrasound features are shared across different TI-RADS classifications, including hypoechogenicity, irregular margins, microcalcifications, and the taller-than-wide feature, which refers to a lesion with a greater anteroposterior dimension than transverse dimension on ultrasound imaging. 

On the basis of the FNAB result, the lesion can be assigned to one of six categories according to the Bethesda system, which provides the framework for further therapeutic decision-making, including qualification for surgical treatment.

The objective of this study was to evaluate the diagnostic value of the taller-than-wide (TTW) ultrasound feature and the parameters describing the dimensions of focal thyroid lesions (height, height-to-width ratio, and height-to-width product) in assessing the risk of belonging to higher Bethesda categories.

## 2. Materials and Methods

A total of 367 patients with focal thyroid lesions qualified for fine-needle aspiration biopsy (FNAB) were included in this retrospective observational study. FNAB specimens were collected at the Center for Pathomorphology of the National Medical Institute of the Ministry of Internal Affairs and Administration in Warsaw. The analysis was based on archival data obtained from the medical records. All data were analyzed in an aggregated and anonymized form.

Ultrasound examinations were performed as part of routine clinical assessment using high-resolution ultrasound equipment by clinicians experienced in thyroid imaging. Nodule dimensions were measured in both transverse and longitudinal planes. The taller-than-wide (TTW) feature was defined as an anteroposterior diameter greater than the transverse diameter measured in the transverse plane.

The identified thyroid lesions were classified into six categories according to the Bethesda System for Reporting Thyroid Cytopathology. For the purposes of the analysis, lesions classified as Bethesda categories I and II were considered low-risk lesions, whereas lesions classified as Bethesda categories III–VI were considered lesions requiring further clinical evaluation and potential surgical management. In particular, Bethesda category V was considered suspicious for malignancy, whereas Bethesda category VI was considered malignant.

The study was approved by the Bioethics Committee at the National Medical Institute of the Ministry of the Interior and Administration in Warsaw (decision No. 62/2025).

### Statistical Analysis

The normality of continuous variables was assessed using the Shapiro–Wilk test. Variables with a normal distribution were described using mean and standard deviation, whereas variables with a non-normal distribution were described using median and interquartile range (Q1–Q3). For categorical variables, the frequency distribution of each category was presented with absolute numbers and percentage values for each category. The results are presented in the table, and the distributions of continuous variables are presented as box plots.

The normal distribution of the data was assessed using the Shapiro–Wilk test, and the homogeneity of variance was assessed using the Bartlett test. Comparisons between groups were made using the Mann–Whitney U test. Variables were tested for monotonic correlations using Spearman’s rank correlation coefficient. This value of the latter ranged from −1 to 1; the following classification of correlation strengths was adopted: |r| = 0—no correlation, 0 < |r| ≤ 0.3—very weak correlation, 0.3 < |r| ≤ 0.5—weak correlation, 0.5 < |r| ≤ 0.7—moderate correlation, 0.7 < |r| ≤ 0.9—strong correlation, 0.9 < |r| < 1.0—very strong correlation, and |r| = 1—full correlation. The correlation results are also presented in the form of heat maps.

The next step in the analysis consisted in the modeling of lesions requiring further clinical evaluation and potential surgical management by means of logistic regression, with nodule height being used as the explanatory variable. Due to the difficulties with interpretation of logistic models, odds ratios (ORs) were calculated for the explanatory variable along with respective 95% confidence intervals (CIs). The effectiveness of the model was then evaluated using the ROC curve and the area under the curve (AUC) value (the higher the value, the better the model fit). The measures of predictive accuracy of the model were also determined:

- Sensitivity—the percentage of positive diagnoses among all truly positive cases;
- Specificity—the percentage of negative diagnoses among all truly negative cases;
- NPV (Negative Predictive Value)—the rate of truly negative cases among all those indicated as negative by the model;
- PPV (Positive Predictive Value)—the rate of truly positive cases among all those indicated as positive by the model.

The cut-off point value determined from the ROC analysis defined the nodule height required for the lesions requiring further diagnostic evaluation. The statistical significance level was defined as α = 0.05, with statistically significant differences falling within the *p* < 0.01 and *p* < 0.001 ranges being additionally marked. Values lower than 0.001 were always given as “*p* < 0.001”. All statistical analyses were performed using R software, version 4.3.2 (R Foundation for Statistical Computing, Vienna, Austria; https://www.r-project.org/).

## 3. Results

The mean age of the study participants (male and female) was 65.2 years (SD = 13.6). The lesions were classified according to the Bethesda system into six categories. Of these, the most common was Class II (57.5%). Class I accounted for 18.8% of cases, and Class III accounted for 12.5%. The shares of each of the other three classes did not exceed 6%.

For further analysis, the lesions were divided into two groups: low-risk lesions (Bethesda categories I and II, 76.3%) and lesions requiring further clinical evaluation and potential surgical management (Bethesda categories III–VI, 23.7%). The characteristics of the study population are presented in Table 1.

Each focal lesion was described by two dimensions of width and height; the quotients and the products of both these values were also calculated.

The mean and the median lesion height were 16.95 mm (SD = 8.65) and 15 mm, respectively. The mean and the median lesion width were 14.15 mm (SD = 6.84) and 13 mm, respectively. The mean taller-than-wide (TTW) ratio was 1.25 (SD = 0.35) while the mean product of both dimensions was 288.94 mm^2^ (SD = 292.82). The characteristics of the lesions are shown in Table 2.

**Figure 1:**
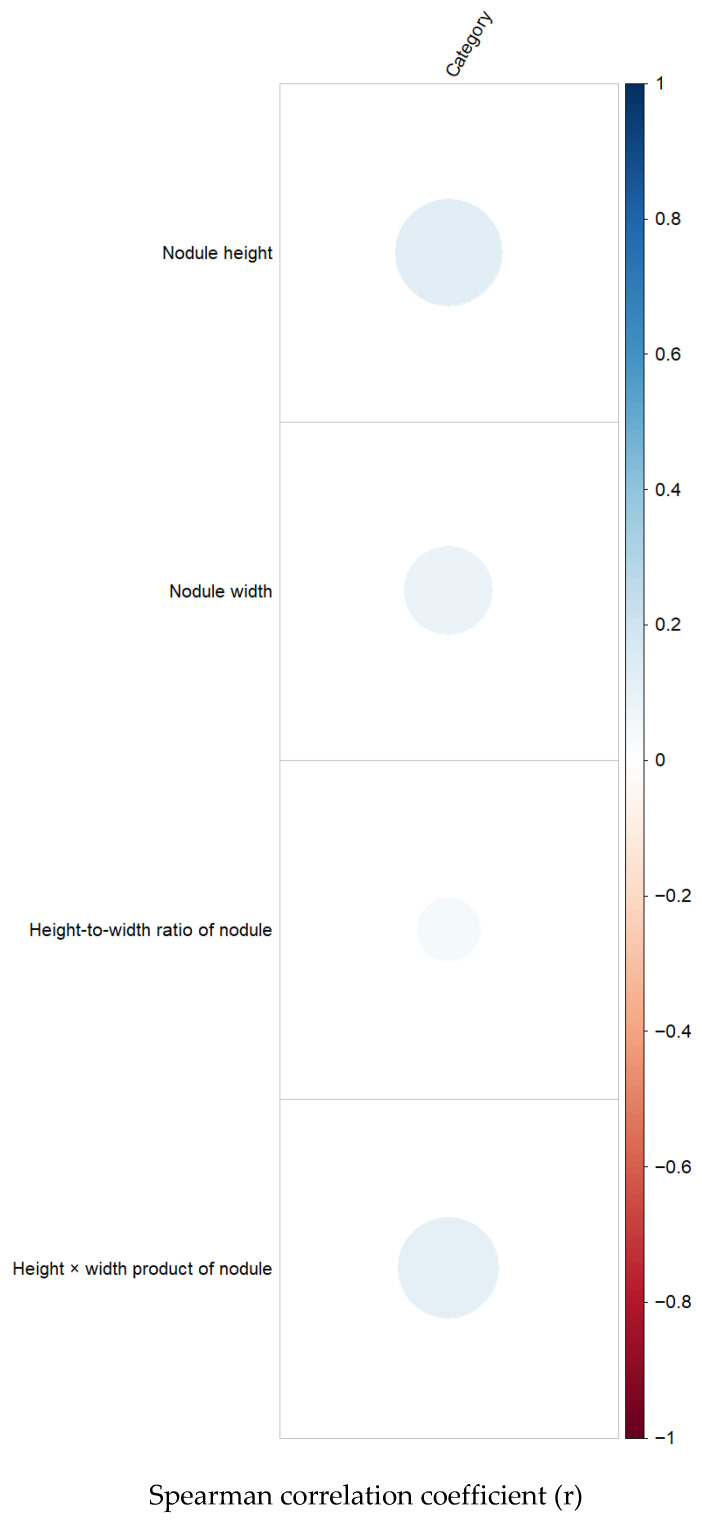
Heat map of Spearman rank correlation coefficients between Bethesda category and ultrasound lesion dimensions.

The Spearman’s rank correlation analysis revealed a positive, statistically significant relationship between the Bethesda class and the height of the focal lesion (r = 0.124; *p* = 0.017) as well as the height and width product value (r = 0.111; *p* = 0.033). No significant relationships were observed between the Bethesda class and lesion width (r = 0.086; *p* = 0.101) or height-to-width ratio (r = 0.044; *p* = 0.405). Detailed results are shown in Table 3 and a visualization of correlations is shown in Figure 1.

**Figure 2:**
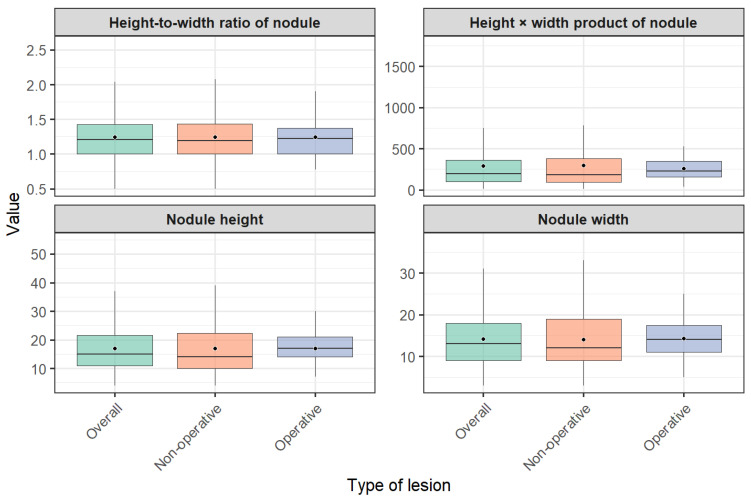
Box plots of nodule dimensions in conservatively and surgically managed lesions. Boxes represent median and interquartile range; whiskers indicate range.

No statistically significant differences were found in comparative analysis between low-risk lesions and lesions requiring further clinical evaluation and potential surgical management in terms of width (*p* = 0.128), height-to-width ratio (*p* = 0.939) or height-by-width product value (*p* = 0.056). A small-effect difference was found for the height dimension (*p* = 0.043; r = 0.106). A summary of the parameters is shown in Table 4, and the box-plot distribution of values is shown in Figure 2.

The logistic regression analysis revealed no significant effect of height-to-width ratio on the risk of a lesion being qualified for surgery (OR = 1.03; 95% CI: 0.52–2.06; *p* = 0.931). The model results are shown in Table 5.

**Figure 3:**
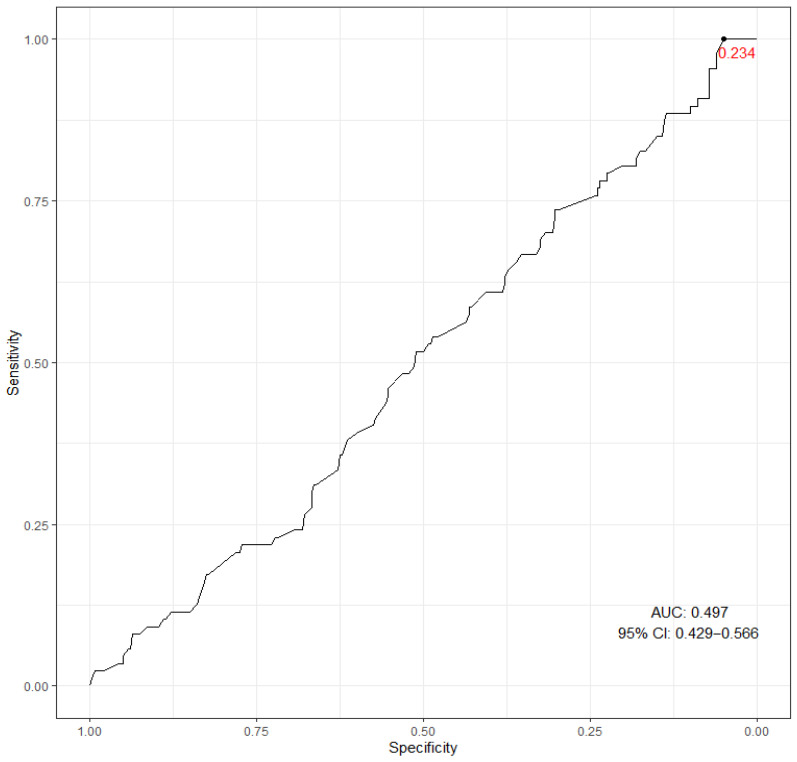
ROC curve of the logistic regression model. The red value (0.234) indicates the probability threshold selected for classification.

The effectiveness of the model was further verified using the ROC curve (Figure 3).

The model presented with low discriminatory ability (AUC = 0.497; 95% CI: 0.429–0.566). The probability threshold of 0.234 corresponded to a height-to-width ratio of 0.772 and had a high sensitivity (100%) with low specificity (5%).

## 4. Discussion

In contrast to the results obtained herein, numerous studies indicate the high value of the TTW feature in assessing the malignancy of focal thyroid lesions. In numerous international publications, this feature is described as significantly associated with malignancy [13,14,15,16,17,18]. According to Li et al. [14], who had analyzed a total of 982 focal thyroid lesions, the taller-than-wide feature had a high discriminatory value with AUC = 0.849. The parameter was distinguished by its high sensitivity and specificity, confirming that the more vertical nature of the lesion favors its interpretation as malignant.

Mattingly et al. [15] showed that higher height-to-width ratios remained associated with higher percentages of malignant lesions; in addition, the authors pointed to the higher diagnostic value of multilevel models compared to the classic binary methodology. The analysis by Huang et al. [16] focusing on lesions less than 1 cm in size revealed a significantly increased odds ratio (OR = 8.99) for the TTW parameter, confirming its crucial diagnostic significance. The authors also emphasized that the taller-than-wide feature was clinically more significant in patients younger than 45 years. In our study, a positive yet weak correlation was observed between the dimensions and the Bethesda class of the focal lesion, with the logistic regression model showing no statistically significant relationship between the height-to-width ratio and the risk of the lesion being qualified for surgery. This discrepancy with the literature data may be due to different anatomical features of the analyzed population, differences in sample size, different approaches to the definition of TTW, as well as the way the study was conducted and the experience of the researchers [8,19,20,21,22].

The taller-than-wide (TTW) feature is considered one of the major ultrasound characteristics associated with higher cytological risk and an increased likelihood of malignancy in previous studies, and is therefore included in several risk stratification systems, including EU-TIRADS. Although our results did not demonstrate a statistically significant association between the height-to-width ratio and the qualification of lesions for surgical management, the observed positive correlation with higher Bethesda categories may still support the clinical relevance of TTW in everyday ultrasound practice. Therefore, TTW should be interpreted together with other ultrasound features and cytological findings rather than as an isolated predictor of higher-risk Bethesda categories or confirmed malignancy [23,24].

The use of dedicated software in quantitative analyses has reduced differences between individual observations in TTW determination, increasing the reliability of these studies [21]. Multimodal approaches, including elastography and contrast-enhanced examinations, further increase diagnostic sensitivity and specificity [25,26]. Published analyses and studies confirm that integration of ultrasound findings with cytologic results translates to more expedient surgical decisions [27,28]. A number of other ultrasound features suggesting an increased risk of focal lesion malignancy, such as irregular outline, deep hypoechogenicity, microcalcifications, or features of extrathyroidal extension, should also be taken into consideration when performing a US scan. These parameters should be analyzed together with the TTW feature when qualifying lesions for fine needle aspiration biopsy or surgical treatment [29,30,31]. A large population-based study involving 8806 patients subjected to ultrasound examination published by Smith-Bindan et al. [20] revealed that the assessment of isolated features suggestive of a higher risk of malignancy can lead to high false-positive rates and higher numbers of unnecessary biopsies, while a combination of two or more features significantly increases the reliability of the diagnosis. Data presented by Wu et al. [21] suggest that software-based assessment of TTW-like lesions had higher reproducibility, as confirmed by the higher κ value relative to the clinicians’ measurements. Interpretation of the data leads to the conclusion that a wider use of IT tools should be considered in routine clinical practice. The need for continuous improvement of systems like EU-TIRADS is also emphasized so as to increase the quality of assessments obtained in routine ultrasound practice [10,11].

The literature on the importance of the taller-then-wide feature and the dimensions of the lesion consistently points to the TTW shape of the lesion seen on ultrasound as being a highly specific marker of a malignant tumor. The clinical problem, however, involves the test’s sensitivity of inference, as literature reports differ in its assessment. The usefulness of inferring from the TTW feature is mentioned in combination with other ultrasound features. Moreover, most studies emphasize the importance of standardization of the measurement techniques. Despite the advances in risk stratification systems that include the TTW feature and the dimensions of the lesion, the variability of malignancy rates in different populations and clinical settings underscores the need for improved, context-specific diagnostic criteria. A critical analysis and synthesis of literature reports is presented in Table 6.

## 5. Study Limitations

An important limitation of this study was its retrospective nature, potentially resulting in selection bias and limiting the control of confounding factors. Another limitation is the relatively small size of the study group, which may limit the generalizability of the findings to the broader population.

In addition, postoperative histopathological results were not available for the study population. Therefore, the reference standard used in the analysis was cytological classification according to the Bethesda system rather than postoperative histopathological examination. The obtained results should be interpreted with caution, as cytological assessment does not always correspond to the final histopathological diagnosis.

Furthermore, the logistic regression model included only the height-to-width ratio and did not account for potential confounding variables, as additional demographic and ultrasound characteristics were not available in the dataset. Future prospective studies should incorporate multivariable analyses to better assess the independent predictive value of the TTW feature. Therefore, the results of the logistic regression should be interpreted as exploratory and should not be considered evidence of the independent predictive value of the TTW feature.

Another important limitation is that the study population consisted exclusively of patients who had already been referred for fine-needle aspiration biopsy. Therefore, the cohort represents a preselected clinical population rather than an unselected sample of thyroid nodules. This selection bias may have influenced the distribution of Bethesda categories and contributed to the limited diagnostic performance observed for the TTW feature, particularly in the ROC analysis.

## 6. Research Perspectives

Further studies should employ prospective research designs involving larger populations and standardized ultrasound assessments; in addition, the use of IT tools, including artificial intelligence (AI)-based decision-making systems, should be expanded to improve the classification process.

## 7. Conclusions

1. Morphometric features of focal lesions, such as their height and the height by width product, showed a positive yet very weak correlation with the Bethesda class; as such, they may be used as auxiliary indicators in cytological risk assessment, as their value as independent predictive factors remains limited.
2. In this cohort, no significant association was observed between the height-to-width ratio and higher-risk Bethesda categories. Therefore, the TTW feature should be interpreted together with other ultrasound characteristics and cytological findings rather than as an isolated predictor.
3. Differences between the results obtained in this study and the reports in international literature may be due to the different size and characteristics of the population, different definitions of the TTW feature, and the varied experiences of individuals performing ultrasound examinations. This suggests the need for continued improvement of classification systems and expansion of the applicability of IT systems in ultrasound scanning.

## Figures and Tables

**Table 1:** Clinical characteristics of the study population and distribution of Bethesda System categories in thyroid nodules (N = 367).

Variable	Parameter	Total (N = 367)
Class	N	367
	Average (SD)	2.2 (1.1)
	Median (Q1–Q3)	2 (2, 2).
	Range	1–6
Bethesda Classification	1	18.8% (N = 69)
	2	57.5% (N = 211)
	3	12.5% (N = 46)
	4	5.4% (N = 20)
	5	2.7% (N = 10)
	6	3% (N = 11)
Type of change	Unresectable	76.3% (N = 280)
	Resectable	23.7% (N = 87)
Age [years]	N	367
	Average (SD)	65.2 (13.6)
	Median (Q1–Q3)	68 (55–75).
	Range	21–94

SD, standard deviation; Q1–Q3, first and third quartiles.

**Table 2:** Comparison of dimensions of lesions of different Bethesda classes.

Variable	Parameter	Total (N = 367)	1 (N = 69)	2 (N = 211)	3 (N = 46)	4 (N = 20)	5 (N = 10)	6 (N = 11)
Nodule height		367	69	211	46	20	10	11
	Average (SD)	16.95 (8.65)	16.12 (9.56)	17.19 (9.51)	16.5 (5.16)	18.35 (4.36)	16.6 (6.06)	17.18 (4.45)
	Median (Q1–Q3)	15 (11–21.5).	13 (9–23).	15 (10.5–22).	16 (13–20.5).	17.5 (15.75–21).	18 (12.5–19.75).	17 (13.5–19).
	Range	4–55	5–53	4–55	7–30	10–27	7–26	13–28
Width of the nodule		367	69	211	46	20	10	11
	Average (SD)	14.15 (6.84)	14.12 (8.66)	14.1 (6.95)	14.26 (4.71)	15.45 (4.58)	13 (4.16)	13.45 (4.95)
	Median (Q1–Q3)	13 (9–18).	12 (7–20).	13 (9–18.5).	14 (11–18).	14 (12–18).	13.5 (10.75–14.75).	12 (10.5–15.5).
	Range	3–38	3–38	3–38	5–24	9–29	5–19	7–25
The ratio of the height to the width of the nodule		367	69	211	46	20	10	11
	Average (SD)	1.25 (0.35)	1.21 (0.33)	1.26 (0.35)	1.22 (0.37)	1.23 (0.29)	1.32 (0.39)	1.35 (0.34)
	Median (Q1–Q3)	1.21 (1–1.43).	1.14 (0.94–1.33).	1.25 (1–1.45).	1.16 (0.95–1.36).	1.26 (1.11–1.31).	1.38 (0.96–1.52).	1.18 (1.1–1.64).
	Range	0.5–2.6	0.82–2.33	0.5–2.6	0.78–2.6	0.8–2	0.78–1.89	1–1.9
The product of the height and width of the nodule		367	69	211	46	20	10	11
	Average (SD)	288.94 (292.82)	301.91 (354.42)	297.26 (315.99)	251.26 (138.4)	296.1 (155.99)	228.8 (117.74)	247.27 (166.22)
	Median (Q1–Q3)	195 (100–360).	156 (70–420).	187 (99–364).	224 (154–374.25).	270 (180–341.25).	249.5 (153.75–325.25).	209 (149.5–278.5).
	Range	12–1786	15–1749	12–1786	42–660	120–783	35–380	91–700

SD, standard deviation; Q1–Q3, first and third quartiles.

**Table 3:** The vector of Spearman’s rank correlation coefficients with the respective *p*-values.

Variable	Correlation with Bethesda Category (r; *p*-Value)
Nodule height	0.124 (0.017)
Nodule width	0.086 (0.101)
Height-to-width ratio	0.044 (0.405)
The product of the height and width of the nodule	0.111 (0.033)

r, Spearman’s rank correlation coefficient.

**Table 4:** Comparison of ultrasound lesion dimensions between surgically treated and conservatively managed patients (N = 367).

Variable	Parameter	Total (N = 367)	Total Number: N = 280	Total Number: N = 87	Test	*p*-Value	Effect Size
Nodule height		367	280	87	Mann–Whitney’s U	0.043	0.106
	Average (SD)	16.95 (8.65)	16.92 (9.52)	17.02 (4.98)			
	Median (Q1–Q3)	15 (11–21.5).	14 (10–22.25).	17 (14–21).			
	Range	4–55	4–55	7–30			
Nodule width		367	280	87	Mann–Whitney’s U	0.1283	0.079
	Average (SD)	14.15 (6.84)	14.1 (7.39)	14.29 (4.64)			
	Median (Q1–Q3)	13 (9–18).	12 (9–19).	14 (11–17.5).			
	Range	3–38	3–38	5–29			
Height-to-width ratio		367	280	87	Mann–Whitney’s U	0.9387	0.004
	Average (SD)	1.25 (0.35)	1.24 (0.35)	1.25 (0.35)			
	Median (Q1–Q3)	1.21 (1–1.43).	1.2 (1–1.43).	1.22 (1–1.38).			
	Range	0.5–2.6	0.5–2.6	0.78–2.6			
The product of the height and width of the nodule		367	280	87	Mann–Whitney’s U	0.0555	0.100
	Average (SD)	288.94 (292.82)	298.4 (325.23)	258.48 (143.31)			
	Median (Q1–Q3)	195 (100–360).	180 (90–378).	225 (155–348.5).			
	Range	12–1786	12–1786	35–783			

SD, standard deviation; Q1–Q3, first and third quartiles.

**Table 5:** Parameters of the statistical analyses performed.

Logistic Regression Results Evaluating the Effect of the Height-to-Width Ratio on the Risk of a Lesion Being Qualified for Surgery						
**Variable**		**Estimate**	***p*** **-value**	**OR**	**LCI**	**UCI**
Height-to-width ratio of nodule		0.03	0.931	1.03	0.517	2.06
Logistic regression model verification parameters						
**Group**	**AUC (95% CI)**	**Threshold**	**Sensitivity**	**Specificity**	**PPV**	**NPV**
Overall	0.497 (0.429, 0.566).	0.234	1.000	0.050	0.246	1.000
Nodule height-to-width ratio corresponding to the probability threshold at the highest sensitivity						
**Probability threshold**			**Height-to-width ratio of nodule at threshold**			
0.234			0.772			

**Table 6:** Critical synthesis of literature on the usefulness of the taller-than-wide (TTW) feature in assessing the risk of thyroid malignancy.

Area	Strengths	Weaknesses
Diagnostic accuracy of the TTW feature	Numerous studies confirm the TTW shape as a highly specific marker of thyroid malignancy, with specificity consistently reported as high across different cohorts and nodule sizes [19,29,32]. The TTW feature is particularly valuable in small nodules and subcentimeter lesions, where it shows strong predictive value in combination with other features [16,32,33]. Machine learning approaches have further validated vertical orientation (TTW) of the lesion as the most predictive feature, improving diagnostic performance [33].	According to previous studies, despite its high specificity, the TTW feature exhibits low sensitivity, which limits its use as an independent marker of malignancy [23,26]. The variability in sensitivity values reported across studies suggests that a significant proportion of malignant tumors can be missed when relying solely on the TTW feature [19,34]. In addition, the TTW feature is less reliable for follicular carcinoma as it is often missing in this type of malignancy [19].
Measurement methodologies and probe orientation	Studies show that the transverse ultrasound plane is generally sufficient and suitable for the assessment of the TTW feature, with longitudinal or combined planes being of minimal added diagnostic value [23,24]. The effect of probe tilt and orientation on TTW assessment is negligible, which supports the use of standard imaging protocols [19]. Software-based quantitative analysis of the TTW feature improves reliability and reduces operator-dependent variability [21].	Discrepancies between clinician and software assessments of the TTW feature were highlighted in some studies, pointing to the possibility of misclassification due to subtle measurement differences or confounding ultrasound features [21]. The lack of universal consensus on the measurement planes and criteria may contribute to inconsistent application of ultrasound measurements in clinical practice [34].
Integration with other ultrasound features and risk stratification systems	Combining the TTW feature with other ultrasound features, such as microcalcifications, hypoechogenicity, and irregular margins, significantly improves the prediction of malignancies and the accuracy of risk stratification [27,29,35]. The modified TIRADS and TTW-weighing systems show improved diagnostic performance and clinical utility [26,33,36]. The inclusion of TTW in multiparametric models facilitates better specification of biopsy indications and a reduction in the number of unnecessary procedures [37,38].	The heterogeneity of risk stratification systems and the variability of TTW weights across the available guidelines complicate direct comparisons and clinical decision-making [39,40]. Some systems may perform more poorly in specific populations or subtypes of tumors, with the optimum combination of features remaining to be standardized [41]. In addition, the risk of malignancy associated with TTW nodules varies significantly between primary/secondary and tertiary care settings, reflecting selection bias and pre-test probability differences [39,42].
Relationship between lesion dimensions and malignancy risk	Studies confirm that the lesion dimensions, especially the height-to-width ratio, are crucial in assessing malignancy, with higher ratios correlating with increased risk [15,32]. Smaller TTW nodules show higher predictive accuracy for papillary thyroid cancer [32]. Combining lesion size with TTW and other features increases sensitivity and specificity [16,25].	There is an established inverse correlation between nodule size and the TTW shape, which complicates risk assessment in larger nodules [29]. Some studies indicate that the TTW feature is less predictive in nodules greater than 1 cm, suggesting size-dependent diagnostic thresholds [32]. The effect of lesion dimensions on malignancy risk is not uniform across various histological types, limiting the generalizability of findings [19].
Impact of clinical conditions and patient selection	Prospective studies in iodine-deficient areas and primary care settings reveal lower malignancy rates in TTW nodules compared to retrospective analyses in tertiary centers, indicating the influence of patient selection and referral bias [39,42]. This underscores the need to contextualize TTW results and pre-test risk in relation to the clinical environment.	The variability in the incidence of malignant neoplasms in different clinical settings questions the external validity of some of the results and may lead to an over- or underestimation of the predictive value of TTW [39]. Limited data are available on the prognostic value of TTW in unspecified cytologic categories and various patient populations, limiting the diagnostic applicability of the feature [41,43].
Methodological robustness and data quality	Large multicenter studies and meta-analyses provide solid evidence supporting the diagnostic role of TTW, with significant sample sizes and histopathological confirmations [19,44]. The use of machine learning and software-based assessments increases objectivity and reproducibility [21,33].	Many studies followed a retrospective design, which has inherent limitations, such as selection bias, variable ultrasound operator knowledge, and inconsistent reference standards [27,45]. The lack of uniform protocols for acquiring and interpreting ultrasound scans contributes to the heterogeneity of results [23,34]. Some studies were based on small sample sizes or focused on specific subgroups, limiting generalizability [31].

## Data Availability

The datasets generated and/or analyzed during the current study are not publicly available due to data protection and privacy regulations but are available from the corresponding author on reasonable request.
